# Uses of Phage Display in Agriculture: Sequence Analysis and Comparative Modeling of Late Embryogenesis Abundant Client Proteins Suggest Protein-Nucleic Acid Binding Functionality

**DOI:** 10.1155/2013/470390

**Published:** 2013-07-09

**Authors:** Rekha Kushwaha, A. Bruce Downie, Christina M. Payne

**Affiliations:** ^1^Agricultural Science Center, Department of Horticulture, University of Kentucky, Lexington, KY 40546, USA; ^2^Seed Biology Group, University of Kentucky, Lexington, KY 40546, USA; ^3^Plant Science Building, Department of Horticulture, University of Kentucky, Lexington, KY 40546, USA; ^4^Department of Chemical and Materials Engineering, University of Kentucky, Lexington, KY 40506, USA; ^5^Center for Computational Sciences, University of Kentucky, Lexington, KY 40506, USA

## Abstract

A group of intrinsically disordered, hydrophilic proteins—Late Embryogenesis Abundant (LEA) proteins—has been linked to survival in plants and animals in periods of stress, putatively through safeguarding enzymatic function and prevention of aggregation in times of dehydration/heat. Yet despite decades of effort, the molecular-level mechanisms defining this protective function remain unknown. A recent effort to understand LEA functionality began with the unique application of phage display, wherein phage display and biopanning over recombinant Seed Maturation Protein homologs from *Arabidopsis thaliana* and *Glycine max* were used to retrieve client proteins at two different temperatures, with one intended to represent heat stress. From this previous study, we identified 21 client proteins for which clones were recovered, sometimes repeatedly. Here, we use sequence analysis and homology modeling of the client proteins to ascertain common sequence and structural properties that may contribute to binding affinity with the protective LEA protein. Our methods uncover what appears to be a predilection for protein-nucleic acid interactions among LEA client proteins, which is suggestive of subcellular residence. The results from this initial computational study will guide future efforts to uncover the protein protective mechanisms during heat stress, potentially leading to phage-display-directed evolution of synthetic LEA molecules.

## 1. Introduction

Water is essential for life. Despite this apparent truism, there are organisms that have phases of their life cycle during which they can withstand dehydration to less than 5% water content on a fresh weight basis. This phenomenon has become known as “anhydrobiosis” or life without water [1, 2]. One of the means by which those organisms capable of anhydrobiosis are thought to retain viability at very low moisture content is through the vitrification of the cytoplasm upon water removal [3, 4]. The cytoplasmic phase transitions, from liquid to viscous to glass, are thought to increasingly impede deleterious biochemical reactions while progressively dampening respiration [5]. A second requirement is to protect those cellular components, dependent on water to maintain their structure/function, using so-called “water replacement” by specific, non-reducing oligosaccharides [2] which, in conjunction with highly hydrophilic proteins, can also enhance the quality and persistence of the glassy state [6, 7]. A third means is to prevent the aggregation of cellular constituents as water is withdrawn, and the distance between macromolecules diminishes [8, 9]. All of these properties have been assigned to various families of the Late Embryogenesis Abundant (LEA) proteins which were first identified [10] and then named [11] from studies of cotton seed proteins found in the embryo.

The characteristic intrinsically disordered structure and high hydrophilicity of the LEA proteins have been used to argue that they may act in a variety of ways to replace water (or compensate for its loss) in dehydrating tissues [12, 13]. Although there are two known LEA structures [14, 15], many of the proteins belonging to this family are dynamically disordered by design [16–18]. This has reasonably led to difficulties in obtaining structural information despite the use of a variety of techniques [19, 20], temperatures, and additives [17, 21]. Although obtaining crystal structures for most LEAs is not likely in the near future, structures of the preferential LEA client proteins may be estimated through homology modeling [22–24] as the same data allowing client protein identification also permits the identification of the region of the client protein to which the LEAs bind. Understanding which proteins are a particular LEA's preeminent substrates provides insights into those functional processes most at risk for dehydration/thermal damage, suggesting novel ways forward in producing more drought-/heat-resistant species. Identification of hallmarks within the bound regions of LEA client proteins will provide the first clues as to which protein topologies are particularly prone to dehydration/heat damage. We hypothesize the regions require protection, which may be achieved through LEA protein binding.

Here, we report functional insights relative to LEA client proteins from application of sequence analysis and comparative modeling. Our examination focuses on identifying commonalities within the set of 21 putative LEA protein interactions previously identified using phage display [25]. Sequence analysis suggests a common theme among many of the LEA client proteins may be protein-nucleic interaction motifs which may provide clues regarding subcellular residence of the LEA proteins themselves. Homology modeling, where feasible, uncovers several structures, varying both in length and tertiary structure, whose common thread may be related to dynamic and chemical behavior more than structural or sequence similarity.

## 2. Comparative Modeling Methods

**Figure 1:**
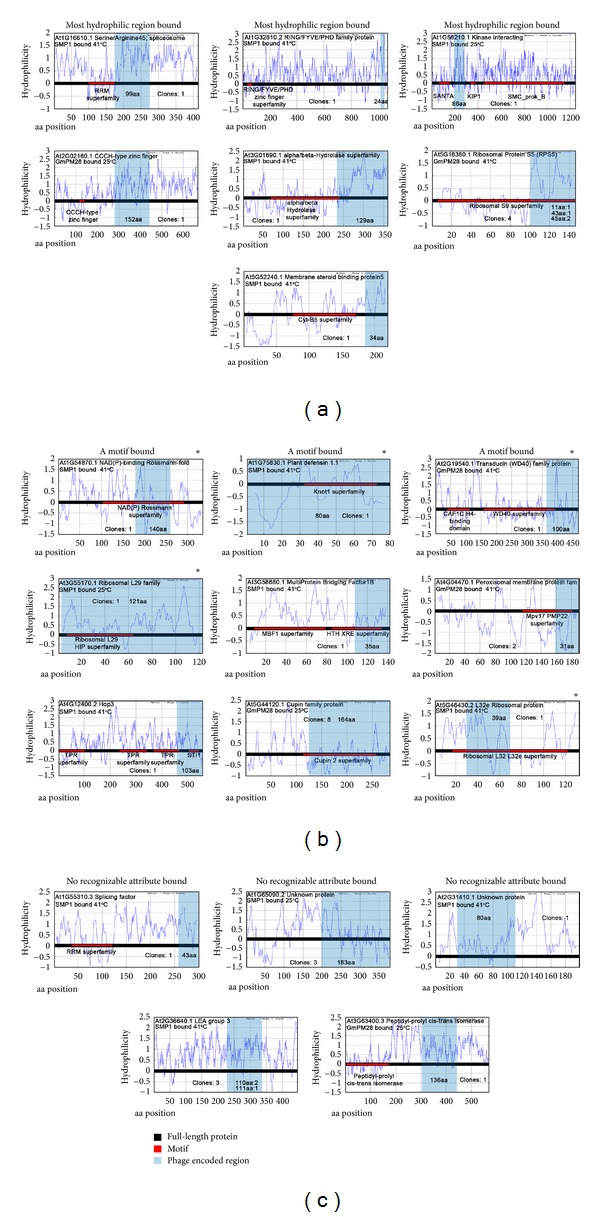
A graphic depiction of the region of the client proteins to which the SMP1 or GmPM28 proteins bound. In each graph, the full-length protein is depicted as a black bar centered at zero on the Hopp/Woods hydrophilicity plot [57] for the protein (retrieved from Expasy Protscale [35]). Recognizable motifs present in the protein are represented as red bars on the black bar under which the Pfam [58] acronym defining the motif superfamily is displayed. The region of the full-length protein displayed on the phage and captured by the LEA is shaded grey. When this region overlaps with a recognizable motif, the protein is assigned to (b) (A motif bound). When it coincides with the most, or among the most, hydrophilic of the proteins regions, it is placed in (a) (or marked by an asterisk in (b)). If the LEA-bound fragment is neither the most hydrophilic nor encoding a recognizable motif, it is placed in (c) (no recognizable attribute bound). In each graph, the size, in amino acids, of the protein moiety bound by the LEA is provided as well as the number of independently acquired clones. If the clones were of different lengths, the number of clones of a specific length is provided. Whether the clone was bound by SMP1 or GmPM28 and the temperature at which the binding occurred are also provided.

Previously, phage display with *Arabidopsis* seed cDNA libraries in T7 phage was used in biopans of recombinant *Arabidopsis thaliana* seed maturation protein 1 (SMP1) and its *Glycine max* homologue, *Gm*PM28 [25] (LEA proteins). Biopanning was performed at 25°C and 41°C to identify proteins potentially involved in induction of secondary dormancy of *Arabidopsis thaliana* as a result of heat stress (see our companion manuscript for a brief synopsis of seed maturation).  Figure 1 illustrates the 21 putative LEA client proteins identified through phage display. The proteins are labeled by the *Arabidopsis* Information Resource (TAIR) locus identifier. Within each plot, the LEA to which the protein binds and the temperature of the biopan are given. These proteins serve as the basis for our sequence analysis and comparative modeling investigation.

The full-length protein sequences to which LEA proteins of the Seed Maturation Protein family bound in phage display [25] were acquired from TAIR. Each protein was used in screens to identify homologs for which suitable three-dimensional (3D) structures had been solved. For the comparative modeling effort, we focused specifically on the regions identified as binding to the LEA homologues. Based on availability of 3D structures similar to these regions, the number of hits was narrowed down to 7 from the original 21 proteins being assessed (AT1G54870.1, AT1G75830.1, AT3G55170.1, AT3G58680.1, AT5G18380.1, AT5G44120.1, and AT5G46430.2).

Homology modeling of the seven LEA client proteins was performed using the Bioinformatics Toolkit from the Max-Planck Institute for Developmental Biology [26]. This suite integrates a number of utilities necessary to complete the modeling process. For each of the seven proteins, HHpred was used to predict secondary structure and sequence homology [27, 28]. HHblits was used to build multiple sequence alignments as input to the homology modeling software [29]. Homology modeling was performed using MODELLER [30].

Each protein used different templates for which the atomic coordinates were obtained from the RCSB Protein Data Bank [31].  Table 1 summarizes the templates used along with a brief description of each. For each of the models, the standard automated MODELLER procedure for structure modeling and optimization was used. This includes the initial rule-based determination of spatial restraints from the alignment and optimization through minimization of restraint violations. Several of the homology models generated include segments other than the bound regions of interest; however, for the purposes of this project, we limit discussion to the phage-display-recovered regions of the LEA client proteins. Visualization of the proteins and templates was accomplished with PyMOL (The PyMOL Molecular Graphics System, Version 1.2r3pre, Schrödinger, LLC.).

Model quality was determined using the Protein Structure and Model Assessment Tool available through the SWISS-MODEL server [32, 33]. The estimated absolute model quality is reported here using the QMEAN *Z*-score in Table 1, which is an estimate related to reference X-ray crystallographic structures [34]. The reported *Z*-scores are standard deviations of the homology model relative to expected values from experimental structures.

## 3. Results and Discussion

Determination of similarity within the LEA client protein subset begins with analysis of similarities both within the bound region and the full-length client protein. For each of the client proteins identified through phage display as described previously [25], Figure 1 illustrates the hydrophilicity profile (Hopp/Woods analysis from ProtScale [35]) of the entire protein along with any identifiable protein domains. The figure has been divided to categorize the client proteins into those binding the most hydrophilic regions, those in which an identifiable protein motif has been bound, and those with no recognizable attributes having been bound. This preliminary analysis and classification of LEA client proteins have overlapping category members (signified by an asterisk, Figure 1). From this analysis, it is not immediately clear what, if anything, this set of proteins has in common. The full-length proteins are wildly variable in length (79–1608 amino acids) as are the regions containing the portion of the proteins to which the LEAs bind (24–183 amino acids). This latter attribute is consistent with the use of random hexamers to synthesize the phage display libraries [36]. Furthermore, the identifiable protein motifs do not appear to have commonality, though several ribosomal proteins appeared to preferentially bind to the LEA proteins. Interpretation of the hydrophilicity profiles is also mystifying, because while often the most or among the most hydrophilic protein regions are recovered via phage display, exceptions exist.

**Figure 2:**
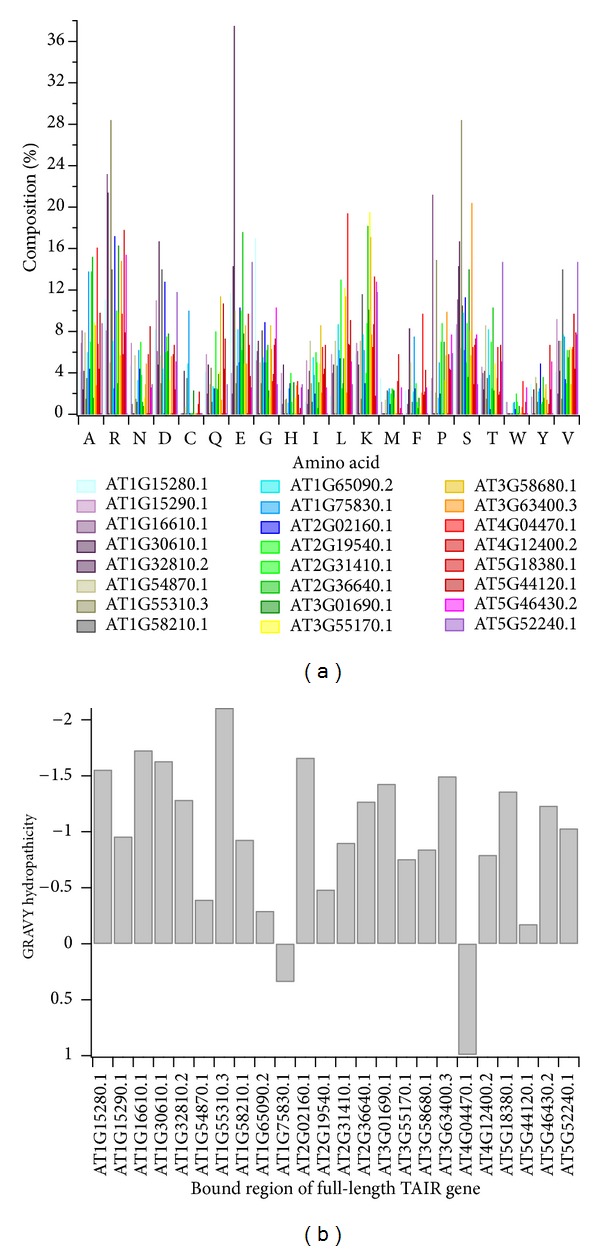
Analysis of the inclusive bound regions of the LEA client proteins identified using phage display. (a) Amino acid composition of the LEA-bound client protein regions is given here by % of the entire individual bound region. The regions are identified by the TAIR locus identifier for the full-length protein, though only composition of the bound region is represented in the plot. (b) A comparison of the GRAVY hydropathicity of the bound regions of the LEA client proteins is given here, again identified by the full-length protein TAIR locus identifier.

Evaluation exclusive to the regions containing those bound by the LEA proteins provides more insight into what the LEA client proteins may have in common. Amino acid composition, normalized by length of the region (Figure 2(a)), reveals that the bound regions have relatively low occurrences of aromatic residues, Phe, Trp, Tyr, and His, and sulfur-containing residues, Cys and Met, lending a general hydrophilicity to the region. Such an attribute would be consistent with the solvent-exposed exterior of a globular protein to which the LEA protein is presumed to bind. Lack of surface-exposed, thiol-containing, amino acids is not surprising given their tendency towards oxidation. Interestingly, the amino acid composition profile is consistent with that of a protein-nucleic acid complex data set examined by Baker and Grant [37]. Baker and Grant postulate that despite the low prevalence of aromatic residues within the binding sites of protein-nucleic acid complexes, aromatic residues still play a critical role in nucleic acid recognition. Relative to our observations, however, the binding site amino acid frequency of protein-nucleic acid complexes appears to be dominated by Arg, Lys, Asn, Glu, Gly, Ser, Thr, and Asp residues, providing us with a common thread potentially linking this set of LEA client proteins.

Further analysis of the hydropathicity of the bound regions provides additional insight into potential functional relationships of the LEA client proteins. The grand average hydropathicity (GRAVY) was determined for each of the LEA client proteins as shown in Figure 2(b). The GRAVY hydropathicity is calculated based on the Kyte and Doolittle [38] hydropathy values for each amino acid, the total of which is subsequently divided by the number of amino acids in the sequence to arrive at an average [35]. A negative value indicates hydrophilicity, and likewise, a positive value indicates hydrophobicity. We see that for a vast majority of our LEA client proteins, the bound region is overwhelmingly hydrophilic, lending credence to the putative role of LEA proteins in the protection of client proteins from dehydration. However, two regions, part of AT1G75830.1 and AT4G04470.1, are identified as hydrophobic, and two others, AT1G65090.2 and AT5G44120.1, are only mildly hydrophilic. For all four of these bound regions, we confirmed that a single residue or subset of residues was not dominating the average, and rather, the hydrophobicity or mild hydrophilicity is indicative of the nature of the entire bound region (see Figure 1).

The entire set of full-length LEA client proteins was also analyzed using WOLF-pSORT (invoking the plant option), a program designed to predict protein localization sites [39]. Of the set, all but three were predicted to reside within a subcellular compartment containing nucleic acid polymers, with most predicted as either nuclear or cytoplasmic. The three outliers in the WOLF-pSORT analysis included AT1G75830.1, AT2G36640.1, and AT5G44120.1. AT1G75830.1 was predicted as extracellular. AT2G36640.1 was predicted to be peroxisomal, and AT5G44120.1 was predicted to be vacuolar. It is noteworthy that two of the three WOLF-pSORT outliers correspond to the hydrophobic or only mildly hydrophilic binding regions. This suggests, as does the amino acid composition, that perhaps an overall commonality of the remaining LEA client proteins is an ability to bind nucleic acids or at the very least promote interaction.

The amino acid sequences of the full-length LEA client proteins were analyzed using two separate protein motif/pattern and signature identification utilities with the aim of uncovering unifying motifs or functionality within the set.  Table 2 summarizes the motifs and patterns uncovered, delineating between those that belong to the sequence region containing the bound moiety and those belonging to the full-length protein excluding this region. Putative amidation motifs (x-G-[RK]-[RK]) were identified using the patmatmotifs utility within the EMBOSS software suite, which searches amino acid sequences against the PROSITE motif database [40]. PROSITE defines an amidation site (PS00009) as situated at the carboxy terminus of an active peptide in a larger precursor protein at the site of cleavage. Typically, peptidylglycine *α*-amidating enzyme (*α*-AE) can utilize the amino group from the C-terminal glycine in this motif to effect the conversion of the amino acid “x” to an amidated-(CO–NH_2_) rather than a carboxylated-(COOH) terminus [41]. Nearly 60% of the full-length sequences contain at least one amidation domain (25% within the binding regions); however, relevance is difficult to determine at this point given the high natural probability of occurrence of this tetrapeptide sequence.

Using the InterProScan protein signature recognition software, potentially meaningful motifs, though not discernibly mutual, were established. The identification of the microbodies C-terminal targeting signal domain, a tripeptide C-terminal consensus sequence occasionally found in peroxisomal proteins [42], in AT2G36640.1 is consistent with the prediction from WOLF-pSORT of this protein as peroxisomal. This is not unexpected, as pSORT algorithms use the SKL motif as recognition mechanism for peroxisomal proteins. The RGD tripeptide sequence motif was also returned in three separate instances, which is thought to promote binding to integrins and similar proteins [43] and appears to be critical in mediation of cell attachment [44]. The leucine zipper and coiled coil motifs were also repeatedly returned by InterProScan searches. The leucine zipper is a protein-protein motif of *α*-helices that dimerizes to form a coiled coil. The leucine zipper is known to participate in DNA-binding and regulation of gene expression [45], and the coiled coil is suspected to more generally participate in protein-protein interactions [46]. Less often, though interesting, nonetheless, the ATP/GTP A motif was returned by InterProScan. This motif, ATP/GTP A, is a glycine-rich loop sequence connecting a *β*-strand and an *α* helix, which has been identified as a conserved region of ATP- and GTP-binding proteins through observation of crystallographic data [47–52]. The loop region is known to interact with the phosphate groups of nucleotides. Finally, several proteins were identified as ribosomal which, along with RNA in protein-RNA interactions, assemble to form ribosomal subunits [53]. While there does not seem to be a single unifying motif or pattern among the set of phage-display-identified LEA client proteins, there does appear to be a common thread of nucleotide interaction based upon known functionality of the identified patterns and motifs.

**Figure 3:**
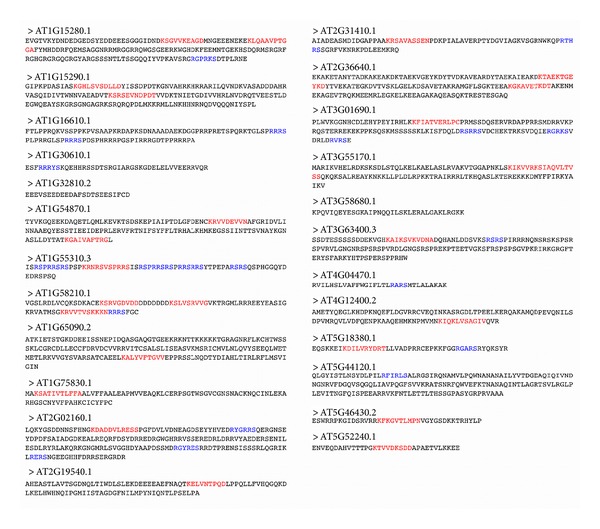
Amino acid sequences of the bound regions of the LEA client proteins. Recurring patterns within the set of sequences have been identified by red and blue text. The red characters indicate the K-x(2,4)-V-x(4)-[ACDGNSTV] pattern. Blue characters indicate the R-x(1,2)-R-x(0,1)-S pattern.

**Figure 4:**
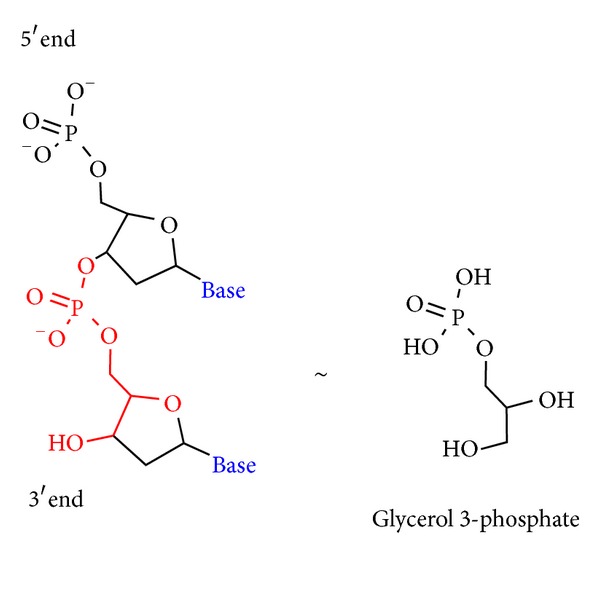
Chemical structure of a nucleotide dimer, left, and glycerol 3-phosphate (glycerophosphate), right. The red lettering on the nucleotide dimer represents the chemical similarity to the glycerophosphate molecule.

Within the amino acid sequences of the bound regions alone, PRATT was used to identify recurring patterns within the unaligned sequences (multiple sequence alignment of the diverse proteins being infeasible) [54].  Figure 3 illustrates the sequences of the bound LEA client protein regions, identified by the TAIR locus identifier. The sequences are coded with red and blue characters according to the two most commonly occurring patterns in the set of proteins. The K-x(2,4)-V-x(4)-[ACDGNSTV] pattern, represented in red, is found in 75% of the bound protein regions identified using phage display. In blue, the R-x(1,2)-R-x(0,1)-S pattern is common to 50% of the bound protein regions. PDBeMotif, a search algorithm providing statistics from 3D structural data, was used to interpret the significance of these two patterns [55]. For both patterns, PDBeMotif suggests—based on existing 3D structural data of proteins containing these sequence patterns—that glycerophosphate, ribose, and deoxyribose are among the structure-bound ligands. These sugars comprise the backbone of nucleic acid, and the presence of the phosphate in glycerophosphate is chemically consistent with an ester-linked phosphate of a nucleotide dimer (Figure 4). While this is by no means confirmation of the common functionality of the LEA client proteins, which can only be guaranteed through experimental means, sequence analysis and pattern/motif algorithms based on existing structural and functional data continually return to the theme of protein-nucleotide interactions.

**Figure 5:**
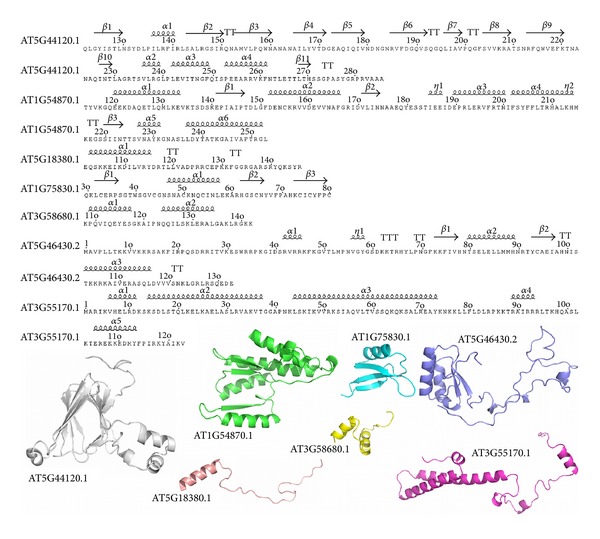
Seven homology models of LEA client proteins, focused on the regions containing the protein moiety to which the LEA proteins bound, were developed. The sequences, numbered by the full-length protein TAIR locus identifier, are shown annotated by secondary structure elements. Secondary structure annotation was accomplished using the ESPript web utility [59]. *β*-Sheets are labeled with a solid black arrow, *α*-helices with medium curly script, *β*-turns with TT, and 3_10_-helices (*η*) with small curly script. Sequence number is also indicated in frequency of ten and corresponds to that of the full-length sequence. Below the sequences, the seven homology models of the bound regions only are shown in cartoon representation. The homology models are labeled, as with the sequences, according to the TAIR locus identifier of the full-length protein to which they belong. The homology model PDB files have been included in Supplementary Materials available online at http://dx.doi.org/10.1155/2013/470390.

The computational analysis of LEA client proteins concludes with homology modeling, where feasible, of the phage-display-bound regions of the LEA client proteins. As with the bioinformatics-based investigation, the intent here was to identify defining characteristics, either structural or chemical, which may yield insight as to why these proteins in particular are consistently returned as LEA-binding partners. Homology modeling methodology and identified structural templates are described in the methods section.. As alluded to the above, we were only able to successfully identify suitable 3D structural templates for seven of the LEA client proteins. Many of the LEA client proteins, including some of those modeled here, exist as membrane-bound proteins and are thus difficult to resolve structurally. The seven LEA client proteins include AT1G54870.1, AT1G75830.1, AT3G55170.1, AT3G58680.1, AT5G18380.1, AT5G44120.1, and AT5G46430.2. We anticipate that as crystallographic methods continue to develop, additional structures will become available to serve as templates to the remaining client proteins. Several other templates were available for portions of the full-length proteins; however, we are restricting this homology modeling study to that within the bound regions, as this should intuitively provide the most information regarding features contributing to the protein-protein interactions.  Figure 5 illustrates the sequences of the seven homology models annotated according to the predicted secondary structure. Below the sequences, cartoon representations of each model are provided.

Structural or sequential representation of these seven protein regions does not provide a striking explanation as to which attribute is acting as a functional link. Several of the structures exhibit relatively large expanses of disordered loop regions, which seem rather uncharacteristic of globular proteins. This is almost certainly related to the hydrophilic nature of the bound regions (Figure 2(a)). We do find it somewhat intriguing, however, that of the three proteins returned by WOLF-pSORT as being neither nuclear nor cytoplasmic, two (AT3G55170.1 and AT1G75830.1) homologous protein structures are available through the Protein Data Bank, though we cannot ascribe significance to this based on the data presented here. With our limited subset of binding site homology models, we can only state that the structures appear to vary significantly from one another and that commonality may lie more in the chemical and dynamical rather than the structural nature of the region.

## 4. Conclusion

A great deal of information relating both sequence and structure of the LEA client protein bound regions and how this contributes to binding remains to be determined. From this initial computational study aiming to shed light on the functional role of LEA proteins through similarity in their bound substrates, we have uncovered what seems to be a predilection for protein-nucleic acid interaction in the LEA client proteins. While this does not yet tell us how the LEA proteins function relative to the bound protein regions, it does suggest hypotheses to be tested concerning the subcellular residence of the LEA proteins under study. An evolutionary relationship between the LEA protein and the substrate protein dictates that the SMP1 and GmPM28 homologs be located in subcellular compartments containing the nucleic acid polymers to which their client proteins apparently bind (i.e., the nucleus, cytoplasm, plastids, and/or mitochondria). Phenotypic consequences for specific LEA protein (or LEA protein family) reductions [36, 56], as well as a demonstration that LEA protein homologs from the Seed Maturation Protein family have preferred client proteins to which they bind [25], suggest that at least some LEA proteins are not redundantly backed up, indiscriminate spacer molecules, and lead to the conclusion that other LEA proteins will also have preferred binding partners. The elucidation of the subfunctionalization of specific LEA proteins concerning which client proteins they bind to is most efficaciously performed using phage display.

In the near term, molecular dynamics simulations of the LEA client protein homology models, including the full-length domains, may provide additional insight into the flexibility and solvation dynamics of the proteins, in addition to directing ongoing experimental phage display efforts. The long-term focus will be on the development of additional homology models as more crystallographic structures become available as well as *de novo* protein design using rapidly developing structure prediction methods. Our continuing aim is the effective integration of computational modeling with phage display for the prediction of protein structures at risk for dehydration or heat damage, uncovering the mechanisms by which LEA proteins perform their protective function. Future endeavors could conceivably encompass phage-display-directed evolution of synthetic LEA proteins engineered to protect labile proteins.

## Supplementary Material

LEA client protein homology models. The seven PDB coordinate files are labeled by the TAIR locus identifier of the full-length protein from which the binding region originates.

## Figures and Tables

**Table 1:** PDB templates used for each of the seven homology models of the LEA client proteins. The four-character PDB identifier is provided. The chain identifier follows the underscore. A brief description of each of the PDB template molecules is provided.

	PDB template	Description	*Z*-score
AT1G54870.1	3ijr_A (no publication)	*Bacillus anthracis* short chain dehydrogenase	−1.24

AT1G75830.1	1ayj_A [60]	*Raphanus sativu*s antifungal protein 1	−0.27

AT3G55170.1	2zkr_v [61]	Mammalian ribosomal 60S subunit	−0.64
	4a17_U [62]	*Tetrahymena thermophilia* 60S ribosomal subunit	
	3u5e_h [63]	Eukaryotic ribosome	
	3iz5_c [64]	*Triticum aestivum* ribosomal protein	

AT3G58680.1	3kxa_A [65]	*Neisseria gonorrhoeae* NGO0477	0.83
	2jvl_A [66]	*Trichoderma reesei* multiprotein bridging factor 1	

AT5G18380.1	3u5c_Q [63]	Eukaryotic ribosome	0.02

AT5G44120.1	3kgl_A [67]	*Brassica napus* 11S globulin, procruciferin	−2.32

AT5G46430.2	4a17_X [62]	*Tetrahymena thermophilia* 60S ribosomal subunit	−0.77

**Table 2:** Patterns and motifs identified using InterProScan and the patmatmotifs utility as part of the EMBOSS package. The full-length protein is identified by the TAIR locus identifier. Patterns and motifs have been separated according to their position either within the binding region or exclusive of the binding region.

	Full-length (excludes binding region)	Binding Region
AT1G15280.1	Amidation motif	Amidation motif
AT1G15290.1	Amidation motif (2) Leucine zipper Coiled coil	Amidation motif
AT1G16610.1	Amidation motif	RGD
AT1G30610.1	RGD ATP/GTP A	—
AT1G32810.2	Zinc finger plant homeodomain Amidation motif (4)	Coiled coil
AT1G54870.1	—	Short-chain dehydrogenase
AT1G55310.3	Amidation motif (2)	—
AT1G58210.1	Coiled coil (11) Leucine zipper	Amidation motif (2)
AT1G65090.2	Amidation motif	—
AT1G75830.1	—	Gamma thionin
AT2G02160.1	Amidation motif Coiled coil	Amidation motif
AT2G19540.1	Amidation motif	—
AT2G31410.1	Coiled coil	—
AT2G36640.1	Microbodies C-ter Coiled coil (2)	Coiled coil
AT3G01690.1	—	Amidation motif
AT3G55170.1	—	Ribosomal L29
AT3G58680.1	Coiled coil	Amidation motif
AT3G63400.3	Prolyl-peptidyl isomerase ATP/GTP A (2) Amidation motif (3)	—
AT4G04470.1	Amidation motif	—
AT4G12400.2	Coiled coil	RGD
AT5G18380.1	Amidation motif Ribosomal	—
AT5G44120.1	11s seed storage	—
AT5G46430.2	—	Ribosomal L32e
AT5G52240.1	—	—
